# PIN1 Modulates Huntingtin Levels and Aggregate Accumulation: An *In vitro* Model

**DOI:** 10.3389/fncel.2017.00121

**Published:** 2017-05-08

**Authors:** Alisia Carnemolla, Silvia Michelazzi, Elena Agostoni

**Affiliations:** International School for Advanced Studies, Area of NeuroscienceTrieste, Italy

**Keywords:** huntingtin, aggregates, PIN1, Huntington's disease, proteasome

## Abstract

Huntington's disease (HD) is a dominantly inherited neurodegenerative disorder characterized by a polyglutamine expansion within the N-terminal region of huntingtin protein (HTT). Cellular mechanisms promoting mutant huntingtin (mHTT) clearance are of great interest in HD pathology as they can lower the level of the mutant protein and its toxic aggregated species, thus affecting disease onset and progression. We have previously shown that the prolyl-isomerase PIN1 represents a promising negative regulator of mHTT aggregate accumulation using a genetically precise HD mouse model, namely *Hdh*^*Q111*^ mice. Therefore, the current study aims at underpinning the mechanism by which PIN1 affects huntingtin's aggregates. We found that PIN1 overexpression led to a reduction of mHTT aggregates in HEK293 cells, and that this could be linked to a negative regulation of mHTT half-life by PIN1. Furthermore, we show that PIN1 has the ability to stimulate the proteasome presenting evidence of a mechanism regulating this phenomenon. Our findings provide a rationale for future investigation into PIN1 with the potential for the development of novel therapeutic strategies.

## Introduction

Huntington's disease (HD) is a progressive, dominantly inherited neurodegenerative disorder that usually manifests in mid-life with psychiatric symptoms, followed by motor impairment and cognitive decline (Papoutsi et al., 2014; Pla et al., 2014; Ross et al., 2014; Zielonka et al., 2015). HD is caused by a CAG triplet repeat expansion within the first exon of huntingtin gene (*HTT*) (HDCRG, 1993), resulting in an expanded polyglutamine (polyQ) segment in the huntingtin protein (HTT). The encoded mutant huntingtin (mHTT) has the propensity to misfold and aggregate (Scherzinger et al., 1997; Gutekunst et al., 1999), producing a whole spectrum of oligomeric species ultimately merging into cellular aggregates and intranuclear inclusions, a major pathological hallmark of HD. Evidence suggests that mHTT aggregation could start off as a coping cellular response, but ultimately, aggregates become co-cause of neuronal dysfunction and cell death (Davies et al., 1997; Difiglia et al., 1997; Gutekunst et al., 1999; Borrell-Pagès et al., 2006; Arrasate and Finkbeiner, 2012). Although the contribution of aggregates to the pathogenesis of HD is not fully understood, the toxicity of soluble monomeric and oligomeric mHTT protein has become a well-accepted evidence (Arrasate and Finkbeiner, 2012) and cellular mechanisms promoting mHTT clearance are of great interest as they could prevent or delay the onset and progression of HD pathology (Sarkar and Rubinsztein, 2008).

mHTT degradation is mediated by two main pathways, the ubiquitin-proteasome system (UPS) (Jana et al., 2005) and autophagy (Sarkar and Rubinsztein, 2008; Koga et al., 2011). Interestingly, ubiquitination can direct HTT for clearance via both pathways (Thompson et al., 2009). Since mHTT accumulations are mainly found in the nuclei of the affected cells in HD *post-mortem* brains (Difiglia et al., 1997), the possibility to enhance the degradative capacities of the UPS, which unlike autophagy operates both in the cytoplasm and the nucleus (Schipper-Krom et al., 2012), may counteract the accumulation of mHTT aggregates.

In a previous study we identified the prolyl-isomerase PIN1 as a promising modifier of some HD phenotypes (Agostoni et al., 2016). PIN1 is a prolyl isomerase, which belongs to the parvulin family, able to catalyze the *cis-trans* isomerization of phosphorylated Ser/Thr-Pro sites (Lu and Zhou, 2007). The conformational change induced by PIN1 has been shown to be central in the modulation of many cellular processes (Lu and Zhou, 2007) and more interestingly, PIN1 dysregulation has been associated with a number of neurodegenerative disorders (Lu et al., 1999a; Pastorino et al., 2006; Ryo et al., 2006; Kesavapany et al., 2007; Lee et al., 2011). Furthermore, several PIN1 substrates have been shown to be targeted for degradation by the UPS upon interaction with PIN1 (Ryo et al., 2007; Nakano et al., 2009; Siepe and Jentsch, 2009; Liou et al., 2011). Interestingly, we have previously shown that genetic *pin1* ablation specifically increased aggregate load in *Hdh*^*Q111*^*::Pin1*^−/−^ mouse striatum (Agostoni et al., 2016). However, the effect of PIN1 on mHTT and the mechanism behind it have remained unknown.

In this study, we provide evidence that PIN1 can negatively regulate the accumulation of mHTT aggregates and propose a mechanism through which PIN1 reduces the level of mHTT. We show that overexpression of PIN1 reduces HTT half-life and consequently, mHTT level leading to a decrease in mHTT aggregate load. We also demonstrate that PIN1 stimulates the activity of the UPS, providing a rationale for future investigations into PIN1 as a potential therapeutic target in HD.

## Materials and methods

### Reagents

Cyclohexamide (CHX) (Sigma, C7698-1G), MG-132 (Sigma, 1211877-36-9), and Epoxomicin (Sigma, 134381-21-8) were solubilised in DMSO according to manufacturer's instructions. CHX was used as inhibitor of protein translation to analyse HTT half-life and was used at a concentration of 40 μg/ml for 2–4 h. MG-132 and Epoxomicin are well-known proteasome blockers; MG-132 was used at a concentration of 10 μM for 6 h and Epoxomicin was used at a concentration of 2.5 μM for 6 h.

### Plasmids and mutagenesis

Httex1Q60GFP in pcDNA3.0 (Invitrogen), encoding the first exon (1–85 aa) of human HTT with 60 glutamines in frame with GFP, was constructed by cloning PCR amplified *HTT* exon1 into EcoRI-XhoI sites of pcDNA3.0GFP vector. The GFP moiety was recovered by XhoI digestion from pGreenLantern-1 (Addgene). htt_1–171_Q21/Q60GFP, encoding the N-terminal 171 amino acids of human HTT, with 21 and 60 glutamines respectively, was constructed as previously described (Persichetti et al., 1999; by subcloning the NcoI-XhoI fragment of *HTT* cDNA into pcDNA3.0GFP vector). The point mutant htt_1–171_Q60S120AGFP was obtained by site directed mutagenesis using two primer sets: for full details on primers sequences see Supplementary Table 1.

HA-PIN1, encoding the human HA tagged PIN1 in pcDNA3.0 vector, was kindly provided by Prof. G. Del Sal (LNCIB, Trieste, Italy); HA-PIN1DM, encoding the human HA tagged PIN1 containing the point mutations Y23A S67E, was constructed by site directed mutagenesis using as template HA-Pin1Y23A in pcDNA3.0-HA, kindly provided by Prof. Del Sal G. (LNCIB, Trieste, Italy) and two new primer sets: for full details on primers sequences see Supplementary Table 1.

pEGFP-C2 was purchased from Clontech Lab. pEYFP^u^ was kindly provided by Prof. Poletti (University of Milan, Milan, Italy); pEYFP was derived from pEYFP^u^ after elimination of the CL1 degron by XhoI-BamHI digestion.

### Cell lines and transfection

HEK293T cells were cultured at 37°C in D-MEM (Dulbecco's modified Eagle's medium), 10% FBS (fetal bovine serum; Sigma, M7524), 100 U/ml penicillin, 100 μg/ml streptomycin (Sigma P0781) and transfected using Lipofectamine 2000 (Invitrogen, 11668019) according to manufacturer's instructions. Transfection efficiency was evaluated by cell count of transfected cells using fluorescent microscopy. For details on transfection efficiency related to each experiment see Supplementary Table 2. Cells were treated with CHX (40 μg/ml, for 2, 3, or 4 h) 6 h after transfection, MG-132 (10 μM for 6 h) 24 h after transfection, or Epoxomicin (2.5 μM for 6 h) 24 h after transfection. SH-SY5Y cells were cultured at 37°C in F12/MEM medium [Ham's F12 (Gibco 31765)/Minimum Essential Media (Sigma)], 15% FBS (Sigma, M7524), 100 U/ml penicillin, 100 μg/ml streptomycin (Sigma P0781), 1% NEAA (Non Essential Amino Acids) and 0.5% Glutamate, and transfected using Lipofectamine 2000 (Invitrogen, 11668019) according to manufacturer's instructions. We evaluated an average of 15% transfection efficiency as estimated by cell count of transfected cells using fluorescent microscopy. Both HEK293 and SH-SY5Y cells were seeded onto 6-well-plates after transfection. Each well of a 6-well plate contained a poly-L lysine treated coverslip that was then used for immunofluorescence experiments; see “Immunostaining and Confocal microscopy” for details on immunofluorescence experiments. For the htt_1–171_Q60GFP + PIN1/PIN1-DM and YFP/YFP^u^ + PIN1/PIN1-DM experiments cells from two wells of a 6-well plate were pooled together to gain the final cell pellet. The cell pellet was then split, half was used for protein analysis via western blotting and the other half was used for mRNA analysis via RT-qPCR.

### Immunostaining and confocal microscopy

For immunostaining, PBS-washed cells were treated as previously described (Trettel et al., 2000). Briefly, cells were seeded onto 13 mm poly-L lysine treated coverslips and allowed to attach for 24 h before transfection. Forty-Eight hours after transfection cells were washed in PBS and fixed in 4% paraformaldehyde for 15 min at RT. After fixation, cells were rinsed in PBS and incubated with 100 mM glycine for 5 min at RT to quench autofluorescence. Membrane permeabilization was performed using 0.1% Triton X-100 for 5 min at RT. Cells were then incubated in 1% BSA for 30 min to block non-specific sites before primary antibody incubation. Both primary and secondary antibodies were incubated in 1% BSA, 1% NGS for 1 h at RT. After primary antibody incubation, cells were washed twice in PBS and subjected to secondary antibody incubation. Nuclei were labeled using DAPI. Cells were washed twice in PBS and mounted on slides using Vectashield (Vector Laboratories) mounting medium. The numbers of the cells expressing transfected DNA and the fluorescent aggregates were manually counted from microscopy captured images. The frequency of aggregates in each transfectant was estimated as a percentage of the numbers of aggregate-positive cells in the cells expressing transfected DNA. Images were captured using Leica confocal microscope TCS SP2, unless otherwise specified. For details on transfection efficiency related to each experiment see Supplementary Table 2.

### Protein extracts, immunoblot analysis, and filter retardation assay

Transfected cells were harvested and lysed in 10% SDS, sonicated for 1 min and heated for 10 min at 95°C. Protein concentration was determined using Bicinconic Acid (BCA) (Thermo Scientific, 23,223, and 23,224). For western blot analysis, 3–10 μg of whole-cell lysates were resolved by SDS-PAGE and transferred onto nitrocellulose membrane. Filter retardation assay was performed as previously described (Huang et al., 1998). Proteins were detected by chemiluminescence following incubation with primary antibodies and horseradish peroxidase-conjugated secondary antibodies; for full details on primary antibodies see Supplementary Table 3.

### Densitometry

Densitometry of western blots was performed using a Bio-Rad GS-800 densitometer and QuantityOne software as previously described (Carnemolla et al., 2014). Developed films were scanned and the average pixel optical density (OD) for each band was measured. The OD of an area devoid of bands was subtracted from the values obtained for bands of interest in order to normalize the OD against background. Relative expression was determined by dividing the normalized OD of bands of interest by the OD of the appropriate loading control for each sample.

### RNA extraction and RT-qPCR

Total RNA was isolated with TRIZOL reagent (Thermo Fischer Scientific, 15596026) according to manufacturer's instructions, quantified by NanoDrop ND-1000 (Thermo Scientific) and analyzed by agarose gel electrophoresis.

Single-strand cDNA was obtained from 1 μg of DNase-treated RNA using iSCRIPT cDNA synthesis kit (Bio-Rad, 1708891) following the manufacturer's instructions. Quantitative PCR reactions were performed with an iCycler iQ instrument (Bio-Rad), using the iQ Custom Syber Green Supermix (Bio-Rad, 4309155). Each reaction was performed in duplicate. Cycle parameters were: 3 min at 95°C (20 s at 95°C, 20 s at 58°C and 30 s at 72°C) for 40 cycles. Specificity of amplicon was determined by melting curve analysis and gel electrophoresis.

Specific forward and reverse primers (Supplementary Table 1) were designed using Beacon Design 5.0 software (Premier Biosoft International). Normalized expression values were calculated using 18S rRNA as endogenous control. YFP and YFPu mRNAs were amplified using EGFP primers.

### Statistical analysis

For tests with only two groups, an unpaired *t*-test was used. For data where four groups were analyzed, such as the CHX experiment, these were analyzed using a two-way ANOVA with treatment and PIN1 construct as between-subject factors. Bonferroni's *post hoc* analysis was applied for multiple comparisons. Statistical analyses were calculated using SPSS Statistics Ver.22 (IBM, Portsmouth, UK). *P*-values of < 0.05 were considered significant. Graphs were constructed using Prism Ver.5.0b (GraphPad Software).

## Results

### PIN1 overexpression reduces mHTT aggregation

We have previously shown that the genetic ablation of *pin1* in *Hdh*^*Q111*^ knock-in mice (Wheeler et al., 1999; *Hdh*^*Q111*^*::Pin1*^−/−^) led to an increase of aggregate load specifically in the striatum of these mice (Agostoni et al., 2016). To investigate the causal relationship between PIN1 expression and mHTT aggregate accumulation we used a short HTT amino-terminal fragment (residues 1–171) bearing a pathogenic glutamine tract (Q60) fused at the carboxy-terminus with a GFP moiety (htt_1–171_Q60GFP; Persichetti et al., 1999).

**Figure 1:**
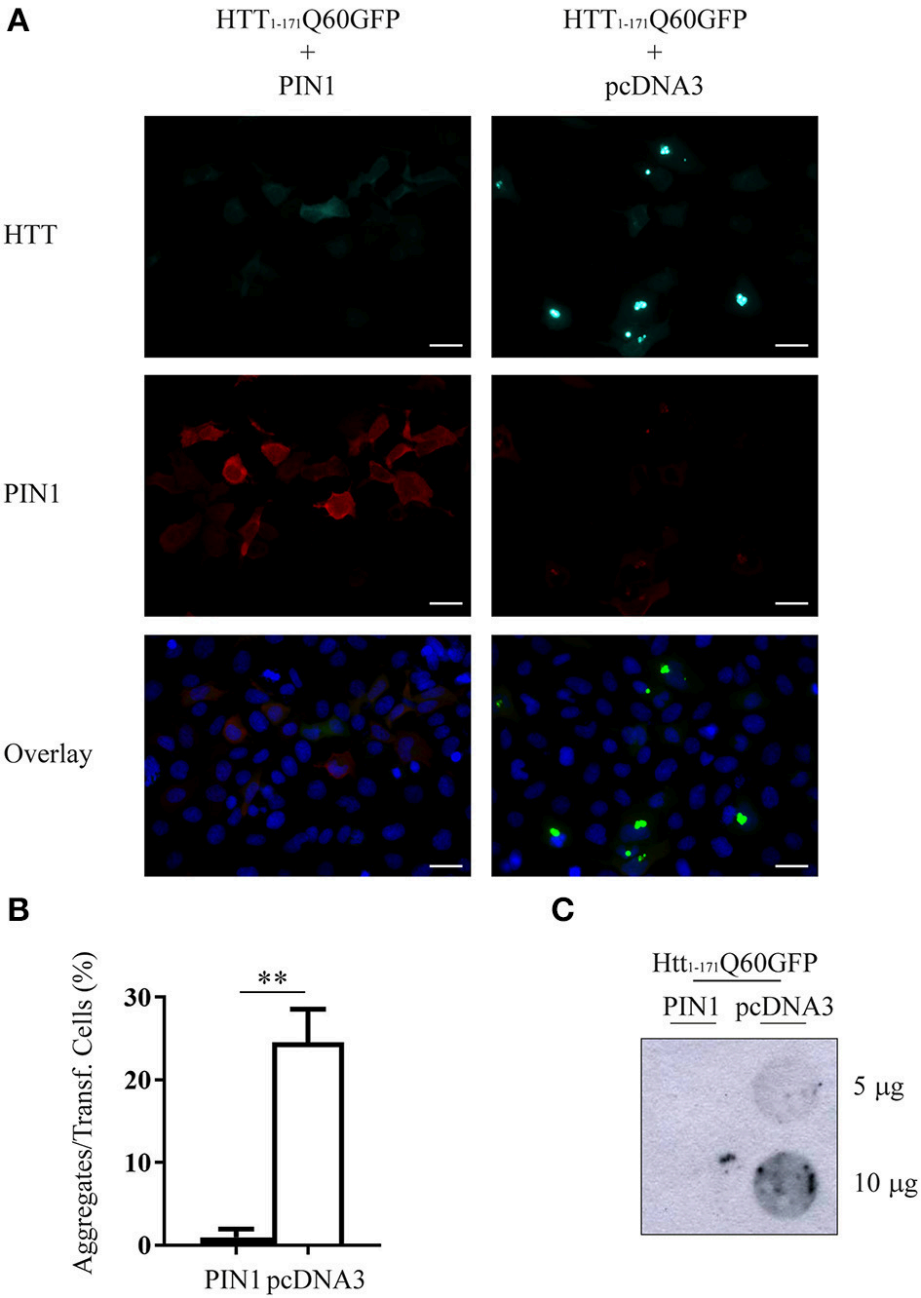
**PIN1 overexpression reduces mHTT aggregate number**. HEK293 cells were co-transfected with htt_1–171_Q60GFP and HA-PIN1 or pcDNA3.0. Cells were harvested for analysis 48 h after transfection. **(A)** Representative immunofluorescent images of co-transfected cells immunostained for PIN1 (anti-HA, red) and counterstained with DAPI (blue). GFP signal (green) represents HTT. Scale bar, 20 μm. Pictures were captured using a Leica CTR 6000. **(B)** mHTT aggregate amount quantified from immunostained cells as shown in **(A)**. **(C)** Representative filter retardation assay of SDS-insoluble aggregates extracted from co-transfected cells. For data on transfection efficiency and total numbers of cells counted see Supplementary Table 2 column “Figure 1.” Data are the mean ± SEM from 3 independent experiments using 3 different batches of cells. ^**^P < 0.01. Asterisk indicates the statistically significant difference in the level of aggregates.

HEK293 cells were co-transfected with htt_1–171_Q60GFP and a construct encoding for human haemagglutinin (HA)-tagged PIN1 (HA-PIN1), or an empty vector (pcDNA3.0-HA) as control. Forty-eight hours after transfection, the proportion of aggregates-containing cells was evaluated by fluorescent microscopy. In line with the data produced in *Hdh*^*Q111*^*::Pin1*^−/−^ mice, but conversely acting, co-expression of PIN1 significantly reduced the number of cells containing mHTT aggregates as compared to the negative control (Figures 1A,B). We also evaluated the presence of SDS-insoluble aggregates by filter retardation assay and we failed in detecting any insoluble mHTT material in the presence of PIN1 (Figure 1C).

It is very well documented that mHTT aggregation rate increases with the length of the polyQ tract (Georgalis et al., 1998; Chen et al., 2002). The expression of an N-terminal mHTT fragment (aa 1–171) containing a longer stretch of glutamines (htt_1–171_Q150GFP) resulted in aggregate formation already 48 h after transfection (Supplementary Figure 1A). We calculated that only ~6% of co-transfected cells presented htt_1–171_Q150GFP aggregates in the presence of PIN1, whereas up to 60% of cells showed visible aggregates in the control (Supplementary Figure 1B). These results suggest that the effect mediated by PIN1 is independent of the length of the polyQ tract.

**Figure 2:**
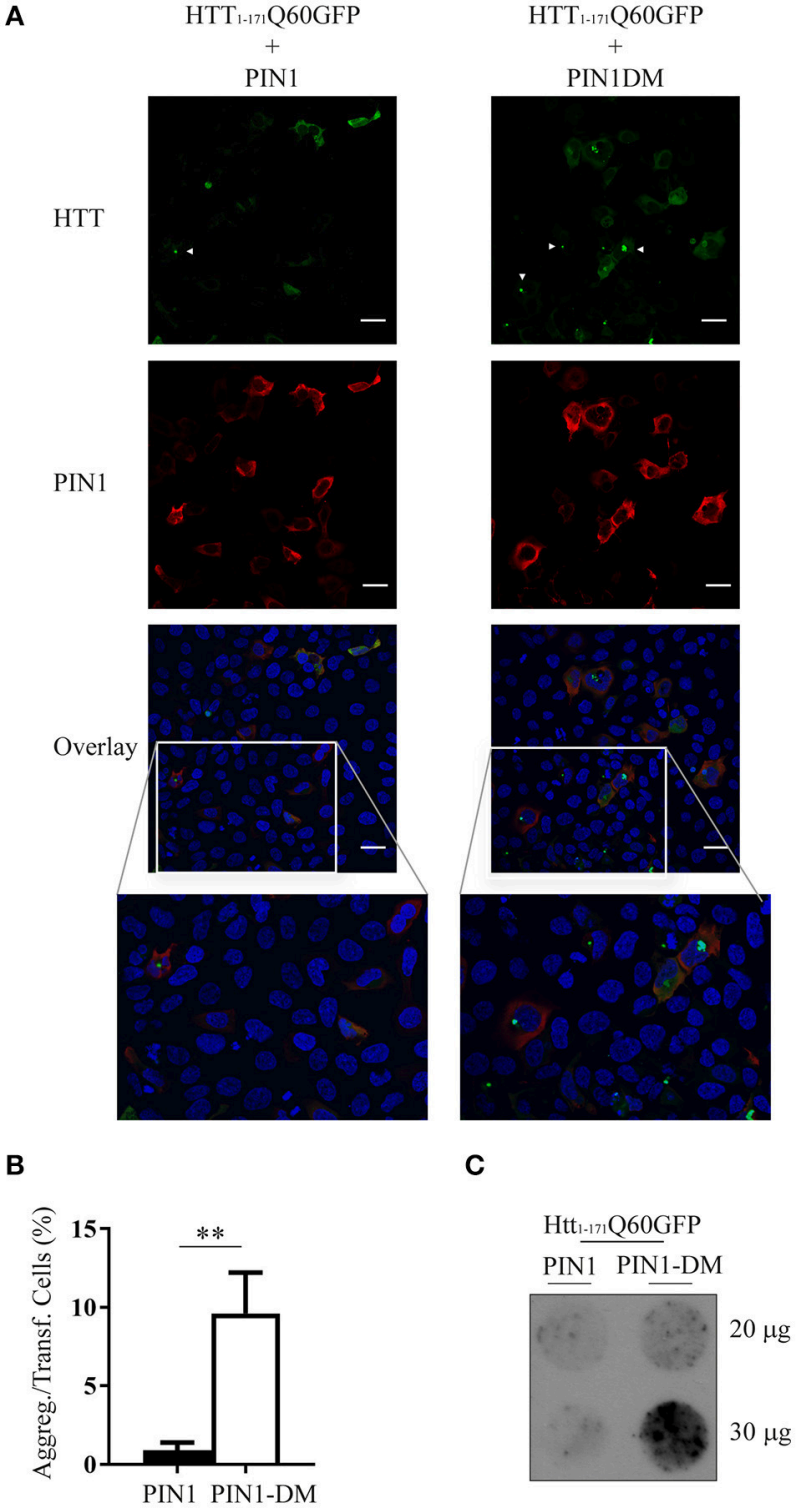
**mHTT aggregate reduction is linked to PIN1 activity**. HEK293 cells were co-transfected with htt_1–171_Q60GFP and HA-PIN1 or HA-PIN1DM. Cells were harvested for analysis 48 h after transfection. **(A)** Representative immunofluorescent images of co-transfected cells immunostained for PIN1 (anti-HA, red) and counterstained with DAPI (blue). GFP signal (green) represents HTT. Scale bar, 20 μm. **(B)** mHTT aggregates amount quantified from immunostained cells as shown in **(A)**. **(C)** Representative filter retardation assay of SDS-insoluble aggregates extracted from co-transfected cells. For data on transfection efficiency and total numbers of cells counted see Supplementary Table 2 column “Figure 2.” Data are the mean ± SEM from 4 independent experiments using 2 different batches of cells. ^**^P < 0.01. Asterisk indicates the statistically significant difference in the level of aggregates. Arrowhead indicates aggregates.

To confirm that the decrease in mHTT aggregates was specifically mediated by PIN1 isomerase activity we inserted two point-mutations into PIN1 coding sequence to generate PIN1^Y23A;S67E^ double mutant (HA-PIN1DM), which is unable to bind its phosphorylated substrates and consequently to catalyze the isomerisation reaction (Lu et al., 1999b; Behrsin et al., 2007). HEK293 cells were transfected with htt_1–171_Q60GFP and HA-PIN1 or HA-PIN1DM; mHTT ability to aggregate was scored by immunofluorescence assay. Consistently, co-expression of PIN1 significantly decreased the number of mHTT aggregates in co-transfected cells, which showed diffuse staining of htt_1–171_Q60GFP, while the expression of the inactive PIN1DM did not affect inclusion accumulation (Figures 2A,B).

Moreover, insoluble mHTT aggregates were detected by filter retardation assay in protein lysates derived from cells co-transfected with PIN1DM, but not with PIN1 (Figure 2C), further supporting PIN1 activity in modulating mHTT aggregation. A cell type-related effect was excluded as similar results were obtained using SH-SY5Y cells in the same co-transfection experiment (Supplementary Figure 2A). SH-SY5Y cells were chosen as representative of a neuronal model in the attempt to mimic more closely what happens *in vivo* in the neurons, as shown by the presence of neuronal intranuclear inclusions rather than perinuclear aggregates (Supplementary Figure 2A).

Altogether, these data suggest that PIN1 may act as a negative regulator of mHTT aggregate accumulation.

### PIN1 overexpression specifically reduces huntingtin protein levels

**Figure 3:**
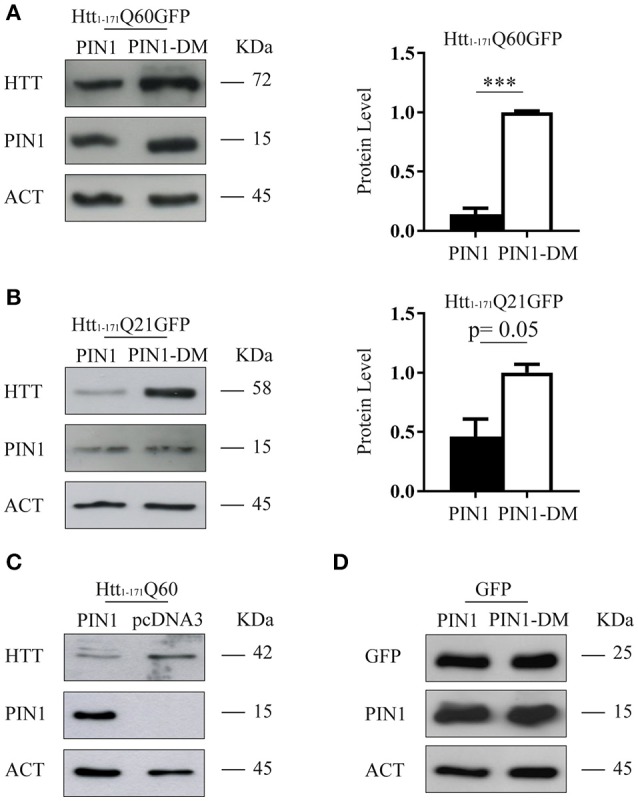
**PIN1 overexpression reduces huntingtin protein levels**. HEK293 cells were co-transfected with HTT-encoding or GFP-encoding plasmids and HA-PIN1 or HA-PIN1DM. Cells were harvested for analysis 48 h after transfection. **(A)** Representative western blot and corresponding protein quantification showing htt_1–171_Q60GFP, PIN1, and β-ACTIN as loading control. **(B)** Representative western blot and corresponding protein quantification showing htt_1–171_Q21GFP, PIN1, and β-ACTIN as loading control. **(C)** Representative western blot showing htt_1–171_Q60, PIN1, and β-ACTIN as loading control. **(D)** Representative western blot showing GFP, PIN1, and β-ACTIN as loading control. Data are the mean ± *SD* from 3 independent experiments. ^***^P < 0.001. Asterisk indicates the statistically significant difference in the level of protein.

The aggregation process of mHTT directly correlates to the length of the polyQ tract, the amount of the mutant protein expressed and the time of exposure of the cell environment to the toxic species, both *in vitro* and *in vivo* (Wanker, 2000; Kaytor et al., 2004). Our experimental design imposes that cells are exposed to the same pathogenic HTT fragment (htt_1–171_Q60GFP) and for the same amount of time (48 h). Therefore, we decided to monitor the level of htt_1–171_Q60GFP protein in the presence of PIN1, or its double mutant, by western blotting. Interestingly, the amount of mHTT was significantly reduced in cells co-expressing PIN1 as compared to the negative control PIN1DM (Figure 3A). Similar results were obtained using SH-SY5Y cells, thus excluding a cell type-related effect (Supplementary Figure 2B). To rule out possible off-target effects of PIN1-DM that could have caused an upregulation of the levels of htt_1–171_Q60GFP, therefore leading to a misinterpretation of the data, we decided to compare the level of htt_1–171_Q60GFP in the presence of PIN1 and PIN1-DM to the level of htt_1–171_Q60GFP in the presence of the empty vector pcDNA3.0 (Supplementary Figure 3). As expected, the overexpression of a second protein, whether PIN1 or PIN1-DM, reduced the level of htt_1–171_Q60GFP as compared to when expressed with pcDNA3.0; nevertheless, the extent of the reduction was much more pronounced in the presence of PIN1, as already shown in Figure 3A. As such, these findings confirmed the absence of any off-target effect of PIN1-DM on the levels of htt_1–171_Q60GFP and suggest that PIN1 activity could affect aggregation by decreasing the amount of soluble mHTT protein.

To investigate whether the effect mediated by PIN1 on mHTT protein was also extended to wild-type HTT (wtHTT) we used a construct encoding for the first 171 amino acids of huntingtin with 21 glutamines fused at the carboxy-terminus with the same GFP moiety (htt_1–171_Q21GFP; Persichetti et al., 1999). Interestingly, the overexpression of PIN1 caused a reduction of wtHTT levels as compared to the control (Figure 3B), suggesting that PIN1 was able to regulate the amount of both wtHTT and mHTT.

To exclude a GFP mediated effect we performed co-transfection experiments using either the N-terminal HTT fragment lacking the GFP moiety (htt_1–171_Q60) or GFP alone. As hypothesized, PIN1 expression caused a reduction of htt_1–171_Q60 protein level (Figure 3C). Interestingly, the levels of GFP protein was not affected by PIN1 (Figure 3D), suggesting that the GFP tag was not the target of PIN1 as well as protein level reduction was not a general consequence of PIN1 overexpression.

Altogether these results suggest that PIN1 is interfering with a cellular process specifically targeting HTT, both wild-type and mutant.

### PIN1 effect is not regulated through direct interaction with huntingtin amino-terminal fragments

Several phosphorylation sites have been identified within HTT protein, including multiple Ser/Thr-Pro motifs that are consensus sequences for PIN1 recruitment (Ehrnhoefer et al., 2011). We have previously shown that the N-terminal fragments htt_1–171_Q21GFP and Htt_1–171_Q150GFP, which contain a single putative PIN1 binding site (huntingtin S_120_–P_121_), were not precipitated by PIN1 in GST-pull down experiments (Grison et al., 2011). To confirm this finding, we decided to use a functional approach to test whether PIN1 activity on HTT protein might directly involve the S_120_–P_121_ site. Using site-directed mutagenesis we generated the mutant construct htt_1–171_Q60S_120_AGFP, where Serine 120 was replaced with Alanine.

**Figure 4:**
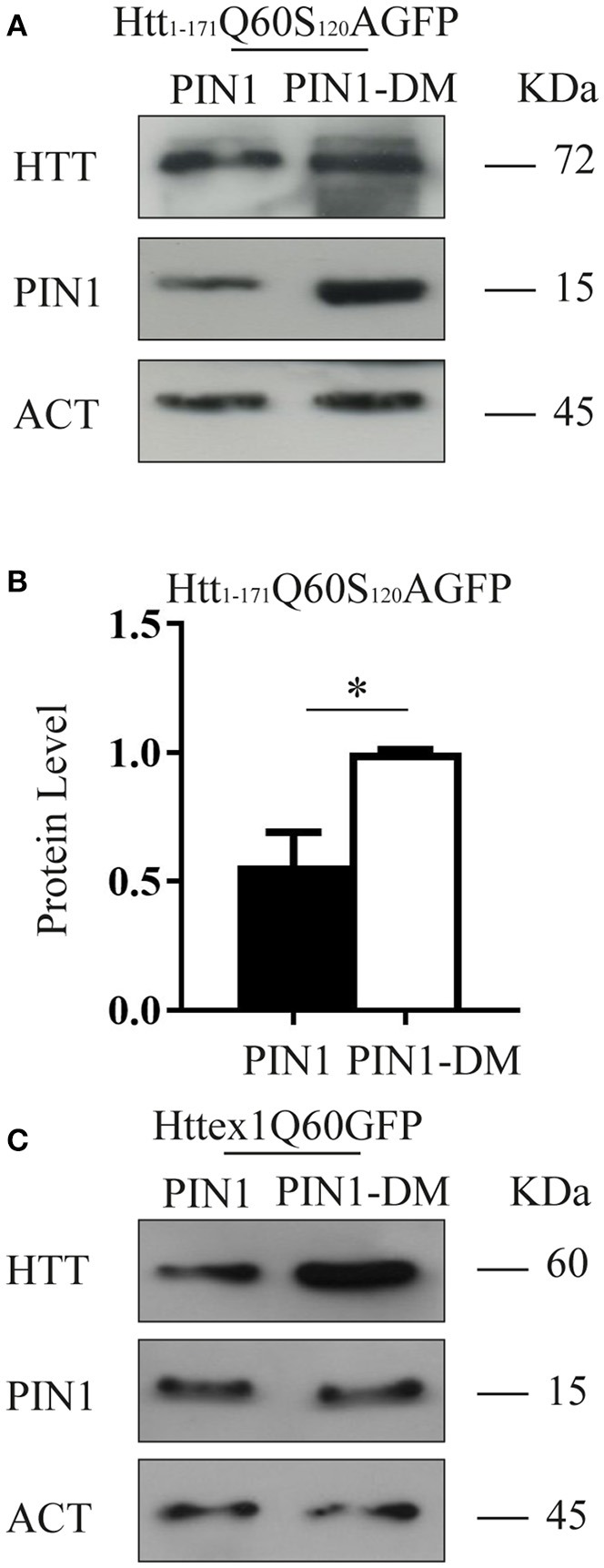
**PIN1 effect is not regulated through a direct interaction with HTT N-terminal fragments**. HEK293 cells were co-transfected with HTT-encoding plasmids and HA-PIN1 or HA-PIN1DM. Cells were harvested for analysis 48 h after transfection. **(A)** Representative western blot showing htt_1–171_Q60S120AGFP, PIN1 and β-ACTIN as loading control. **(B)** Relative protein level quantification of htt_1–171_Q60S120AGFP in the presence of PIN1 as compared to PIN1DM. **(C)** Representative western blot showing httex1Q60GFP, PIN1, and β-ACTIN as loading control. Data are the mean ± *SD* from 3 independent experiments. ^*^P < 0.05. Asterisk indicates the statistically significant difference in the level of protein.

HEK293 cells were co-transfected with htt_1–171_Q60S_120_AGFP and PIN1 or PIN1DM as control. In keeping with the data shown so far, the expression of htt_1–171_Q60S_120_AGFP was significantly reduced by PIN1 as compared to PIN1DM (Figures 4A,B).

To further support this hypothesis we used a shorter mHTT fragment, namely HTT exon 1 (httex1Q60GFP), which does not contain the Ser_120_Pro site. Consistently with our previous data, we observed a reduction in the level of expression of this shorter mHTT fusion protein upon co-expression with HA-PIN1 and not with HA-PIN1DM (Figure 4C). Taken together, these results show that a direct interaction between htt_1–171_Q60GFP and PIN1 is unlikely to be the cause of the observed phenotype.

### PIN1 reduces huntingtin half-life stimulating its degradation through the UPS

**Figure 5:**
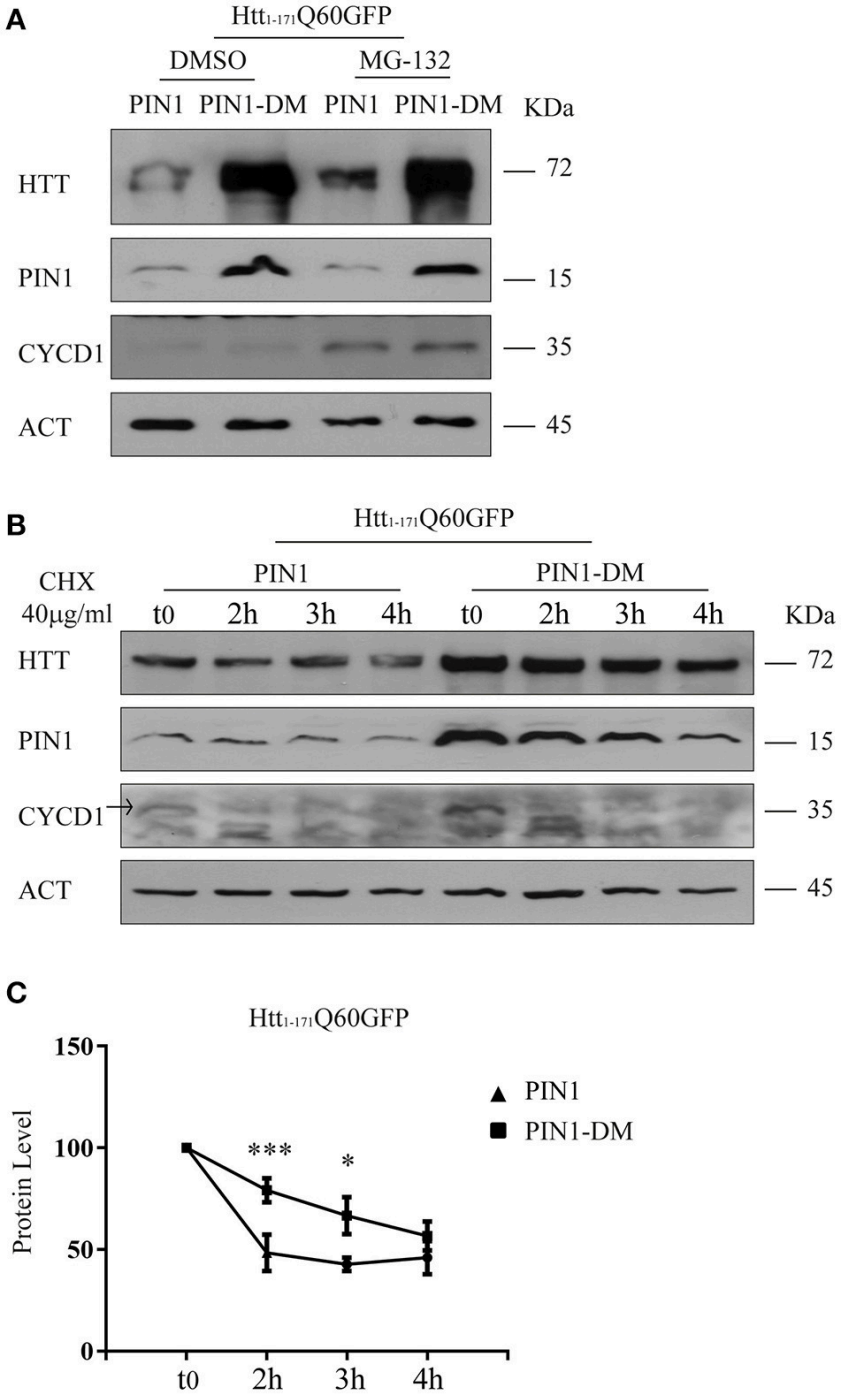
**PIN1 regulates mHTT half-life**. HEK293 cells were co-transfected with htt_1–171_Q60GFP and HA-PIN1 or HA-PIN1DM. Cells were harvested for analysis at indicated time points. **(A)** Representative western blot showing htt_1–171_Q60GFP upon MG-132 treatment (10 μM for 6 h) in the presence of PIN1 as compared to PIN1DM. Western blot also shows PIN1, CYCLIN-D1, and β-ACTIN as loading control. Cells were treated 24 h after transfection. **(B)** Representative western blot showing htt_1–171_Q60GFP levels upon treatment with CHX (40 μg/ml), at different time points, in the presence of PIN1 as compared to PIN1DM. Westerm Blot also shows PIN1, CYCLIN-D1, and β-ACTIN as loading control. CHX treatment started 6 h after transfection. **(C)** Relative htt_1–171_Q60AGFP protein level based on western blots quantification as shown in **(B)**. Data are the mean ± SEM from 6 independent experiments using 2 different batches of cells. ^*^P < 0.05, ^***^P < 0.001. Asterisk indicates the statistically significant difference in the level of protein between PIN1 and PIN1DM-expressing cells at the indicated time point. Values were calculated relative to corresponding t0 sample.

It has been widely documented that N-terminal fragments of HTT are substrates of the proteasome (Jana et al., 2001; Ravikumar et al., 2002; Chandra et al., 2008). To recapitulate these findings, HEK293 cells transfected with htt_1–171_Q60GFP were treated with the proteasome inhibitor MG-132 (10 μM), or DMSO as control, 24 h after transfection. As expected, htt_1–171_Q60GFP accumulated upon proteasome blockade (Supplementary Figure 4A). Hence, we hypothesized that the reduced amount of htt_1–171_Q60GFP might be due to enhanced protein degradation mediated by PIN1. As such, htt_1–171_Q60GFP levels were increased in the presence of PIN1 when proteasome activity was blocked upon MG-132 treatment (Figure 5A). MG-132 treatment also produced a similar increase in the level of endogenous cyclin D1 (CYCD1), an internal control employed to verify the effectiveness of the chemical blocker (Figure 5A). A drug-specific related effect was excluded as similar results were obtained using Epoxomicin (2.5 μM for 6 h), a different proteasome blocker, in the same co-transfection experiments (Supplementary Figure 4B). These results would also suggest that any off-target effect of PIN1-DM on the activity of the UPS can be ruled out as htt_1–171_Q60GFP was able to accumulate in the presence of PIN1-DM upon proteasome blockade as it would have happened if overexpressed alone (Supplementary Figure 4A) or with any other known non-interfering protein.

Conversely to what is observed for htt_1–171_Q60GFP, GFP levels were not altered by PIN1 overexpression (Figure 3D). GFP is a highly stable protein that is not normally degraded by the proteasome (Bence et al., 2001; Verhoef et al., 2002), therefore, we reasoned that PIN1 might be able to stimulate the clearance of htt_1–171_Q60GFP through the UPS. To investigate this hypothesis we evaluated the steady state level of HTT in the presence of PIN1.

HEK293 cells were co-transfected with htt_1–171_Q60GFP and HA-PIN1 or HA-PIN1DM as control. After 6 h, cells were treated with 40 μg/ml of cyclohexamide (CHX), harvested at regular time intervals upon treatment (0, 2, 3, and 4 h) and protein lysates were analyzed by western blotting. Interestingly, 2 h post treatment the relative amount of htt_1–171_Q60GFP was significantly reduced of about 2.5-fold in the presence of PIN1, whereas a reduction of 1.4-fold in the level of mHTT was detected when co-expressed with PIN1DM as compared to t_0_ (Figures 5B,C). A significant difference in the level of htt_1–171_Q60GFP is also observed in the presence of PIN1 at 3 h post treatment as compared to PIN1-DM, but not at 4 h when it is likely that the sensitivity of the technique might be limiting detection and/or the effect of the drug might be fading away. It is important to highlight that CHX treatment started 6 h after transfection when the overall level of htt_1–171_Q60GFP was likely to be very low yet. This condition was specifically sought to be able to exclusively evaluate the level of soluble htt_1–171_Q60GFP before the beginning of any seeding event and oligomer formation. Nevertheless, the low levels of htt_1–171_Q60GFP might have contributed in limiting the sensitivity of the technique in these conditions. Taken together, these results show that PIN1 overexpression reduces the half-life of htt_1–171_Q60GFP protein, suggesting that the mechanism might involve the UPS, and provide a link between PIN1 activity and the reduction in mHTT aggregate load.

To rule out any possible stimulatory effect of PIN1 on other degradative processes that could have accounted for the reduction in the level of htt_1–171_Q60GFP, we decided to analyze autophagy by monitoring the level of BECLIN1 by western blotting. HEK293 cells were transfected with htt_1–171_Q60GFP, or PIN1, or PIN1-DM singularly, or co-transfected with htt_1–171_Q60GFP and PIN1 or PIN1-DM as control. Cells were harvested 24 h after transfection for analysis. Interestingly, we failed in detecting any upregulation of BECLIN1 that would have suggested an increase in autophagosome induced by PIN1, either when transfected alone, or in co-transfection with htt_1–171_Q60GFP (Supplementary Figure 4C). As such, these results suggest that is unlikely that PIN1 can promote the reduction of htt_1–171_Q60GFP levels through a stimulation of the autophagic process, therefore suggesting a central role for the UPS as target of PIN1 activity. In addition, these data confirm once again the absence of any off-target effect of PIN1-DM.

### PIN1 stimulates protein flow through the proteasome

The data shown so far suggest that PIN1 can reduce the amount of mHTT aggregates by negatively affecting the half-life of HTT N-terminal fragments by promoting its degradation via the UPS.

To test whether PIN1 effect was specific for HTT or more widely directed against the degradation process we used the YFP^u^ reporter system (Bence et al., 2001; Bennett et al., 2005). YFP^u^ is normally rapidly degraded by the UPS (t_1/2_ ~30 min; Bence et al., 2001; Bennett et al., 2005; Supplementary Figure 4D); therefore, it represents an appropriate reporter to test the activity of the proteasome in our experimental conditions.

**Figure 6:**
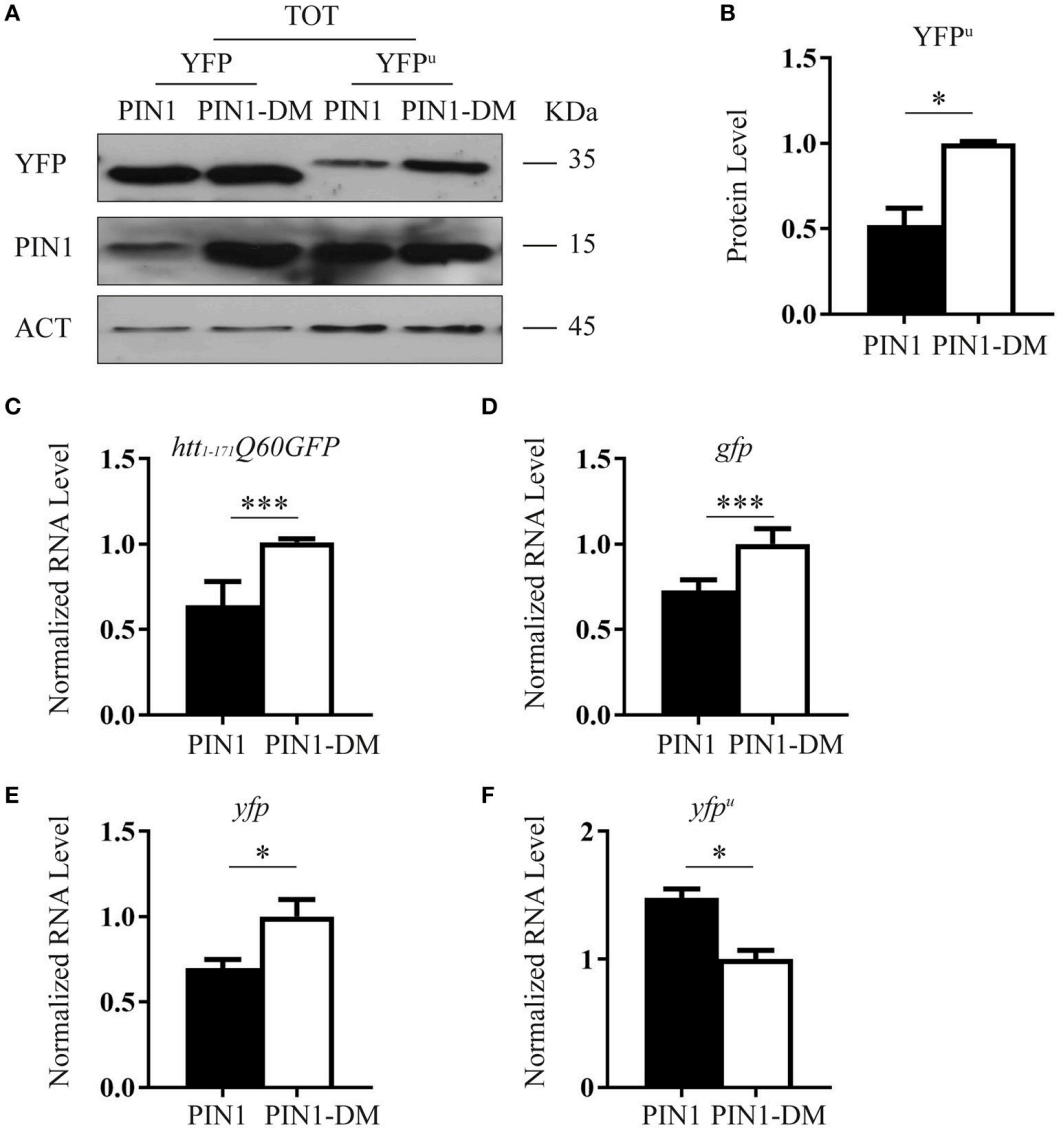
**PIN1 stimulates UPS activity. (A)** HEK293 cells were co-transfected with YFP^u^ or YFP and HA-PIN1 or HA-PIN1DM. Cells were harvested for analysis 48 h after transfection. Representative western blot showing YFP, YFP^u^, PIN1, and β-ACTIN as loading control. **(B)** Relative protein level quantification of YFP^u^ in the presence of PIN1 as compared to PIN1DM. **(C–F)** qPCR analysis of the expression levels of *htt*_1–171_*Q60GFP* **(C)**, *gfp* **(D)**, *yfp* **(E)**, and *yfp*^u^ **(F)** in the presence of PIN1 as compared to PIN1DM. **(C,D)** Data are the mean ± SEM from 4 independent experiments using 2 different batches of cells. **(E,F)** Data are the mean ± *SD* from 3 independent experiments. ^***^P < 0.001, ^*^P < 0.05. Asterisk indicates the statistically significant difference in the level of protein and mRNA.

HEK293 cells were co-transfected with YFP^u^ and HA-PIN1 or HA-PIN1DM. Forty-eight hours after transfection the level of YFP^u^ protein was evaluated by western blot. Interestingly, YFP^u^ signal was significantly reduced in cells co-expressing PIN1 as compared to the negative control (Figures 6A,B).

The same experiment was performed using YFP, a protein known not to be a substrate of the proteasome (Supplementary Figure 4D; Bence et al., 2001; Bennett et al., 2005). As predicted, co-expression of PIN1 did not decrease the level of YFP protein as compared to PIN1DM (Figure 6A).

To rule out the possibility that reduced levels of htt_1–171_Q60GFP and YFP^u^ might account for lower transcription efficiency in the presence of PIN1, we measured mRNA expression levels by RT-qPCR. Indeed, PIN1 has been reported to negatively modulate transcriptional activity of RNA polymerase II (RNAP II) by influencing the phosphorylation status of the C-terminal domain of the largest subunit (Xu et al., 2003; Xu and Manley, 2007). We measured mRNA expression of *htt*_1–171_*Q60GFP, gfp, yfp*, and *yfp*^u^ constructs in transfected cells co-expressing PIN1 or PIN1DM. As expected, a reduction in transcription efficiency was observed in the presence of PIN1 with respect to its negative control. Interestingly, despite the extent of the mRNA reduction was the same between *htt*_1–171_*Q60GFP, gfp*, and *yfp* (Figures 6C–E) we did not detect a corresponding reduction at the protein level for GFP and YFP, but only for htt_1–171_Q60GFP (Figures 3A,D,6A), suggesting that PIN1 effect on the mRNA can be overcome if the protein has a long half-life (i.e., it is not a substrate of the proteasome) and therefore, the degradation pathway followed by the protein of interest might be responsible for the amount of protein detected rather than the amount of mRNA produced. Interestingly, we were not able to detect a downregulation in the mRNA level of *yfp*^u^ in the presence of PIN1 (Figure 6F). These results might suggest that some compensatory mechanisms, such as a more stable *yfp*^u^ mRNA, an event also described for other UPS reporters (Bowman et al., 2005), may counteract the negative effect of PIN1 on the activity of the RNA polymerase II. Furthermore, these findings point to an effect of PIN1 on YFP^u^ exclusively at the protein level further supporting that PIN1 overexpression might increase protein flow through the proteasome.

## Discussion

Protein aggregation has been shown to be a critical mediator of the cell and tissue deterioration that is the characteristic of HD. There is evidence to suggest that mHTT accumulation could start off as a beneficial cellular response, but ultimately, large aggregates and inclusions become co-cause of cell dysregulation and cell death (Arrasate and Finkbeiner, 2012).

In the last decades, research has been focusing on identifying mechanisms to selectively reduce the amount of mHTT in the attempt to remove what have been increasingly considered the most toxic species, i.e., soluble monomeric and oligomeric mHTT (Clabough, 2013). Proteasome impairment has long been considered causative in HD (Finkbeiner and Mitra, 2008) and several studies have shown that mHTT can induce UPS impairment (Bence et al., 2001; Holmberg et al., 2004; Venkatraman et al., 2004). Nevertheless, more recent works have provided evidence of a normally functioning UPS in the presence of mHTT aggregates in different HD models (Bennett et al., 2005; Bett et al., 2006, 2009; Mitra et al., 2009a,b; Ortega et al., 2010). Finally, Ortega et al. demonstrated that mHTT does induce an initial impairment of the UPS that is then recovered when mHTT inclusion bodies emerge (Ortega et al., 2010). On the other hand, evidence suggests that proteasome activity decreases during aging (Saez and Vilchez, 2014). Whether induced by mHTT or caused by age-related proteostasis alterations (Mitra et al., 2009a; Vilchez et al., 2014), reduced processivity of the UPS is a target which amelioration can likely bring strength to a treatment against HD.

In the present study we analyze the role of PIN1 as a negative regulator of mHTT aggregation and provide a mechanism by which PIN1 can reduce the amount of mHTT. Importantly, we show that PIN1 is able to act at the level of soluble mHTT to reduce aggregate load through the stimulation of the UPS.

**Figure 7:**
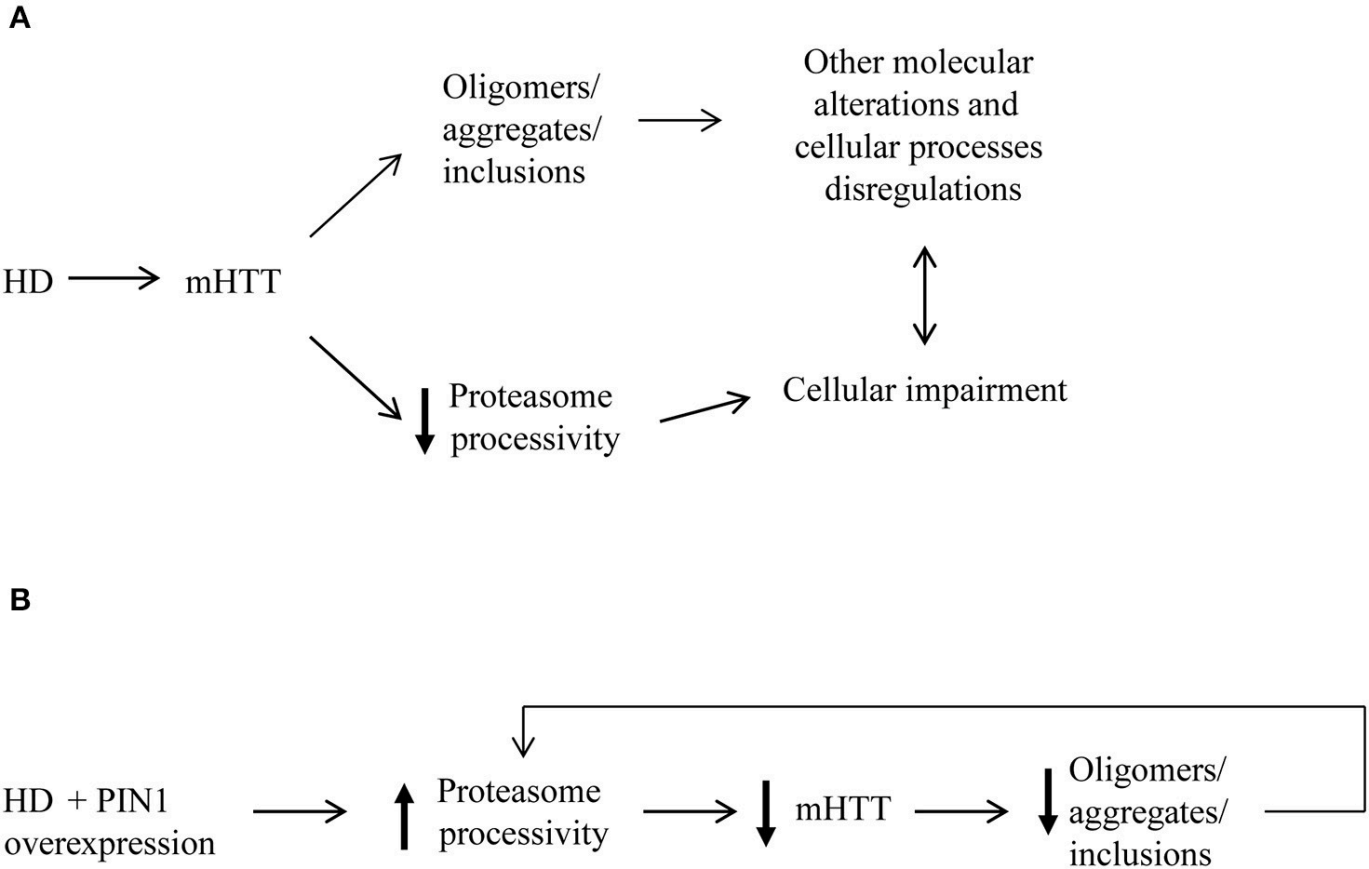
**Proposed model for mHTT aggregate reduction induced by PIN1. (A)** The presence of mHTT aggregates as well as disruption in the protein flow through the proteasome contribute to several alterations of many cellular processes in HD. **(B)** Our data suggest that the overexpression of PIN1 could stimulate proteasome activity leading to an increased degradation mHTT N-terminal fragments. Increased mHTT clearance results in reduced levels of mHTT protein and, as a consequence of this, of mHTT aggregate load. This might create a feedback loop that release some of the pressure on the proteasome as well as other cellular mechanisms targeted by mHTT that could potentially lead to a slowdown of the degenerative processes.

Consistently with our previous *in vivo* data (Agostoni et al., 2016) where we showed that PIN1 ablation specifically increased aggregate load in *Hdh*^*Q111*^*::Pin1*^−/−^ mouse striatum (Agostoni et al., 2016), here we show that PIN1 over-expression reduced mHTT aggregation in a polyglutamine length-independent manner *in vitro*. More interestingly, we observed the ability of PIN1 to reduce the level of soluble HTT by stimulating the activity of the proteasome (Figure 7). Our mRNA data also support a role of the UPS-mediated degradative process as the main target of the effect of PIN1 activity. We detected a significant down-regulation of the mRNA levels of *htt*_1–171_*Q60GFP, gfp*, and *yfp* in line with previously published data (Xu et al., 2003; Xu and Manley, 2007). Interestingly, the mRNA reduction did not reflect into a reduction of the corresponding GFP and YFP proteins, which are very stable proteins and are not normally degraded by the proteasome (Bence et al., 2001; Verhoef et al., 2002; Bennett et al., 2005). In addition, we were not able to detect any negative regulation of the expression of *yfp*^u^ mRNA that could have contributed to the significant reduction of the level of YFP^u^ protein in the presence of PIN1. These findings suggest that, in our experimental conditions, the half-life and the degradation pathway followed by the protein of interest are crucial in determining the amount of protein that is detected, rather than the amount of mRNA that is produced.

It has been proposed that PIN1 might be able to regulate phosphorylation-dependent ubiquitylation of its substrates, therefore modulating protein degradation (Liou et al., 2011). Our data provide evidence to support such hypothesis; the results obtained using the proteasome reporter YFP^u^ showed an overall increase of protein flow through the proteasome when overexpressing PIN1, whereas no effect on autophagy was detected. Although the process might not be specific for mHTT, several evidences suggest that the regulation of intracellular mHTT levels is a coping response and is critical to HD pathogenesis (Finkbeiner and Mitra, 2008; Clabough, 2013). Furthermore, lowering the levels of both wild-type and mHTT to a level not lower than 50% has been shown not to be too detrimental (Yu et al., 2012; Wild and Tabrizi, 2014) as mHTT is able to retain fundamental wild-type functions (Duyao et al., 1995; White et al., 1997; Cattaneo et al., 2001; Reiner et al., 2003). If mHTT was particularly resistant to proteolysis then protein turnover would be delayed; indeed, mHTT may cause a rearrangement of the processing list of UPS substrates taking priority and causing the accumulation of other substrates, without affecting the overall activity rate of the UPS (Finkbeiner and Mitra, 2008). Therefore, a fine titration of PIN1 levels might have a double effect: to stimulate mHTT clearance and to retune the cellular equilibrium back by stabilizing the rate of turnover of other cellular proteins.

We have previously shown (Grison et al., 2011) and confirmed herein with a functional approach that PIN1 does not interact with short N-terminal HTT fragments. As such, the modulation of mHTT half-life could be due to a general effect of PIN1 on proteasomal processivity or we could postulate the presence of a third partner, such as a kinase, which function is regulated by PIN1, able to interact with both PIN1 and HTT to convey the degradation message. This scenario would not be that unlikely as it has been already described in Parkinson's disease where synphilin-1 plays the intermediate role between PIN1 and α-synuclein (Kesavapany et al., 2007).

Despite the down sides that overexpressing a highly interconnected protein such as PIN1 might cause, the possibility to lower the pressure to a system that during the course of the disease is doomed to collapse is extremely appealing and is envisaged might drastically affect the progression of HD.

We conclude that our findings are an encouraging proof of principle that the manipulation of PIN1 can improve disease phenotype in the context of HD. Furthermore, this suggests that a pharmacological alteration of the levels and/or activity of PIN1 could be a promising therapeutic avenue for treatment of HD.

## Author contributions

AC and EA conceived and designed the study, performed experiments, analyzed the data, and wrote the paper. SM provided reagents, materials and analysis tools. All authors discussed the results and commented on the manuscript.

## Funding

This work was supported by SISSA intramural funds. The funders had no role in study design, data collection and analysis, decision to publish, or preparation of the manuscript.

### Conflict of interest statement

The authors declare that the research was conducted in the absence of any commercial or financial relationships that could be construed as a potential conflict of interest.

## References

[B1] Agostoni E. Michelazzi S. Maurutto M. Carnemolla A. Ciani Y. Vatta P.. (2016). Effects of pin1 loss in Hdh(Q111) knock-in mice. Front. Cell. Neurosci. 10:110. 10.3389/fncel.2016.00110 27199664 PMC4852193

[B2] Arrasate M. Finkbeiner S. (2012). Protein aggregates in Huntington's disease. Exp. Neurol. 238, 1–11. 10.1016/j.expneurol.2011.12.013 22200539 PMC3909772

[B3] Behrsin C. D. Bailey M. L. Bateman K. S. Hamilton K. S. Wahl L. M. Brandl C. J.. (2007). Functionally important residues in the peptidyl-prolyl isomerase Pin1 revealed by unigenic evolution. J. Mol. Biol. 365, 1143–1162. 10.1016/j.jmb.2006.10.078 17113106

[B4] Bence N. F. Sampat R. M. Kopito R. R. (2001). Impairment of the ubiquitin-proteasome system by protein aggregation. Science 292, 1552–1555. 10.1126/science.292.5521.1552 11375494

[B5] Bennett E. J. Bence N. F. Jayakumar R. Kopito R. R. (2005). Global impairment of the ubiquitin-proteasome system by nuclear or cytoplasmic protein aggregates precedes inclusion body formation. Mol. Cell 17, 351–365. 10.1016/j.molcel.2004.12.021 15694337

[B6] Bett J. S. Cook C. Petrucelli L. Bates G. P. (2009). The ubiquitin-proteasome reporter GFPu does not accumulate in neurons of the R6/2 transgenic mouse model of Huntington's disease. PLoS ONE 4:e5128. 10.1371/journal.pone.0005128 19352500 PMC2662425

[B7] Bett J. S. Goellner G. M. Woodman B. Pratt G. Rechsteiner M. Bates G. P. (2006). Proteasome impairment does not contribute to pathogenesis in R6/2 Huntington's disease mice: exclusion of proteasome activator REGgamma as a therapeutic target. Hum. Mol. Genet. 15, 33–44. 10.1093/hmg/ddi423 16311253

[B8] Borrell-Pagès M. Zala D. Humbert S. Saudou F. (2006). Huntington's disease: from huntingtin function and dysfunction to therapeutic strategies. Cell. Mol. Life Sci. 63, 2642–2660. 10.1007/s00018-006-6242-0 17041811 PMC11136202

[B9] Bowman A. B. Yoo S. Y. Dantuma N. P. Zoghbi H. Y. (2005). Neuronal dysfunction in a polyglutamine disease model occurs in the absence of ubiquitin-proteasome system impairment and inversely correlates with the degree of nuclear inclusion formation. Hum. Mol. Genet. 14, 679–691. 10.1093/hmg/ddi064 15661755

[B10] Carnemolla A. Labbadia J. P. Lazell H. Neueder A. Moussaoui S. Bates G. P. (2014). Contesting the dogma of an age-related heat shock response impairment: implications for cardiac-specific age-related disorders. Hum. Mol. Genet. 23, 3641–3656. 10.1093/hmg/ddu073 24556212 PMC4065144

[B11] Cattaneo E. Rigamonti D. Goffredo D. Zuccato C. Squitieri F. Sipione S. (2001). Loss of normal huntingtin function: new developments in Huntington's disease research. Trends Neurosci. 24, 182–188. 10.1016/S0166-2236(00)01721-5 11182459

[B12] Chandra S. Shao J. Li J. X. Li M. Longo F. M. Diamond M. I. (2008). A common motif targets huntingtin and the androgen receptor to the proteasome. J. Biol. Chem. 283, 23950–23955. 10.1074/jbc.M800467200 18586675 PMC2527109

[B13] Chen S. Berthelier V. Hamilton J. B. O'nuallain B. Wetzel R. (2002). Amyloid-like features of polyglutamine aggregates and their assembly kinetics. Biochemistry 41, 7391–7399. 10.1021/bi011772q 12044172

[B14] Clabough E. B. (2013). Huntington's disease: the past, present, and future search for disease modifiers. Yale J. Biol. Med. 86, 217–233. 23766742 PMC3670441

[B15] Davies S. W. Turmaine M. Cozens B. A. Difiglia M. Sharp A. H. Ross C. A.. (1997). Formation of neuronal intranuclear inclusions underlies the neurological dysfunction in mice transgenic for the HD mutation. Cell 90, 537–548. 10.1016/S0092-8674(00)80513-9 9267033

[B16] Difiglia M. Sapp E. Chase K. O. Davies S. W. Bates G. P. Vonsattel J. P.. (1997). Aggregation of huntingtin in neuronal intranuclear inclusions and dystrophic neurites in brain. Science 277, 1990–1993. 10.1126/science.277.5334.1990 9302293

[B17] Duyao M. P. Auerbach A. B. Ryan A. Persichetti F. Barnes G. T. McNeil S. M.. (1995). Inactivation of the mouse Huntington's disease gene homolog Hdh. Science 269, 407–410. 10.1126/science.7618107 7618107

[B18] Ehrnhoefer D. E. Sutton L. Hayden M. R. (2011). Small changes, big impact: posttranslational modifications and function of huntingtin in Huntington disease. Neuroscientist 17, 475–492. 10.1177/1073858410390378 21311053 PMC3200085

[B19] Finkbeiner S. Mitra S. (2008). The ubiquitin-proteasome pathway in Huntington's disease. Scientific World Journal 8, 421–433. 10.1100/tsw.2008.60 18454252 PMC2637619

[B20] Georgalis Y. Starikov E. B. Hollenbach B. Lurz R. Scherzinger E. Saenger W.. (1998). Huntingtin aggregation monitored by dynamic light scattering. Proc. Natl. Acad. Sci. U.S.A. 95, 6118–6121. 10.1073/pnas.95.11.6118 9600927 PMC27595

[B21] Grison A. Mantovani F. Comel A. Agostoni E. Gustincich S. Persichetti F.. (2011). Ser46 phosphorylation and prolyl-isomerase Pin1-mediated isomerization of p53 are key events in p53-dependent apoptosis induced by mutant huntingtin. Proc. Natl. Acad. Sci. U.S.A. 108, 17979–17984. 10.1073/pnas.1106198108 22011578 PMC3207660

[B22] Gutekunst C. A. Li S. H. Yi H. Mulroy J. S. Kuemmerle S. Jones R.. (1999). Nuclear and neuropil aggregates in Huntington's disease: relationship to neuropathology. J. Neurosci. 19, 2522–2534. 10087066 10.1523/JNEUROSCI.19-07-02522.1999 PMC6786077

[B23] HDCRG (1993). A novel gene containing a trinucleotide repeat that is expanded and unstable on Huntington's disease chromosomes. The Huntington's Disease Collaborative Research Group. Cell 72, 971–983. 8458085 10.1016/0092-8674(93)90585-e

[B24] Holmberg C. I. Staniszewski K. E. Mensah K. N. Matouschek A. Morimoto R. I. (2004). Inefficient degradation of truncated polyglutamine proteins by the proteasome. EMBO J. 23, 4307–4318. 10.1038/sj.emboj.7600426 15470501 PMC524390

[B25] Huang C. C. Faber P. W. Persichetti F. Mittal V. Vonsattel J. P. Macdonald M. E.. (1998). Amyloid formation by mutant huntingtin: threshold, progressivity and recruitment of normal polyglutamine proteins. Somat. Cell Mol. Genet. 24, 217–233. 10.1023/B:SCAM.0000007124.19463.e5 10410676

[B26] Jana N. R. Dikshit P. Goswami A. Kotliarova S. Murata S. Tanaka K.. (2005). Co-chaperone CHIP associates with expanded polyglutamine protein and promotes their degradation by proteasomes. J. Biol. Chem. 280, 11635–11640. 10.1074/jbc.M412042200 15664989

[B27] Jana N. R. Zemskov E. A. Wang G. H. Nukina N. (2001). Altered proteasomal function due to the expression of polyglutamine-expanded truncated N-terminal huntingtin induces apoptosis by caspase activation through mitochondrial cytochrome c release. Hum. Mol. Genet. 10, 1049–1059. 10.1093/hmg/10.10.1049 11331615

[B28] Kaytor M. D. Wilkinson K. D. Warren S. T. (2004). Modulating huntingtin half-life alters polyglutamine-dependent aggregate formation and cell toxicity. J. Neurochem. 89, 962–973. 10.1111/j.1471-4159.2004.02376.x 15140195

[B29] Kesavapany S. Patel V. Zheng Y. L. Pareek T. K. Bjelogrlic M. Albers W.. (2007). Inhibition of Pin1 reduces glutamate-induced perikaryal accumulation of phosphorylated neurofilament-H in neurons. Mol. Biol. Cell 18, 3645–3655. 10.1091/mbc.E07-03-0237 17626162 PMC1951754

[B30] Koga H. Martinez-Vicente M. Arias E. Kaushik S. Sulzer D. Cuervo A. M. (2011). Constitutive upregulation of chaperone-mediated autophagy in Huntington's disease. J. Neurosci. 31, 18492–18505. 10.1523/JNEUROSCI.3219-11.2011 22171050 PMC3282924

[B31] Lee T. H. Pastorino L. Lu K. P. (2011). Peptidyl-prolyl cis-trans isomerase Pin1 in ageing, cancer and Alzheimer disease. Expert Rev. Mol. Med. 13:e21. 10.1017/S1462399411001906 21682951

[B32] Liou Y. C. Zhou X. Z. Lu K. P. (2011). Prolyl isomerase Pin1 as a molecular switch to determine the fate of phosphoproteins. Trends Biochem. Sci. 36, 501–514. 10.1016/j.tibs.2011.07.001 21852138 PMC3185210

[B33] Lu K. P. Zhou X. Z. (2007). The prolyl isomerase PIN1: a pivotal new twist in phosphorylation signalling and disease. Nat. Rev. Mol. Cell Biol. 8, 904–916. 10.1038/nrm2261 17878917

[B34] Lu P. J. Wulf G. Zhou X. Z. Davies P. Lu K. P. (1999a). The prolyl isomerase Pin1 restores the function of Alzheimer-associated phosphorylated tau protein. Nature 399, 784–788. 10.1038/21650 10391244

[B35] Lu P. J. Zhou X. Z. Shen M. Lu K. P. (1999b). Function of WW domains as phosphoserine- or phosphothreonine-binding modules. Science 283, 1325–1328. 10.1126/science.283.5406.1325 10037602

[B36] Mitra S. Tsvetkov A. S. Finkbeiner S. (2009a). Protein turnover and inclusion body formation. Autophagy 5, 1037–1038. 10.4161/auto.5.7.9291 19838079 PMC2892253

[B37] Mitra S. Tsvetkov A. S. Finkbeiner S. (2009b). Single neuron ubiquitin-proteasome dynamics accompanying inclusion body formation in huntington disease. J. Biol. Chem. 284, 4398–4403. 10.1074/jbc.M806269200 19074152 PMC2640959

[B38] Nakano A. Koinuma D. Miyazawa K. Uchida T. Saitoh M. Kawabata M.. (2009). Pin1 down-regulates transforming growth factor-beta (TGF-beta) signaling by inducing degradation of Smad proteins. J. Biol. Chem. 284, 6109–6115. 10.1074/jbc.M804659200 19122240

[B39] Ortega Z. Díaz-Hernández M. Maynard C. J. Hernández F. Dantuma N. P. Lucas J. J. (2010). Acute polyglutamine expression in inducible mouse model unravels ubiquitin/proteasome system impairment and permanent recovery attributable to aggregate formation. J. Neurosci. 30, 3675–3688. 10.1523/JNEUROSCI.5673-09.2010 20220001 PMC6632247

[B40] Papoutsi M. Labuschagne I. Tabrizi S. J. Stout J. C. (2014). The cognitive burden in Huntington's disease: pathology, phenotype, and mechanisms of compensation. Mov. Disord. 29, 673–683. 10.1002/mds.25864 24757115

[B41] Pastorino L. Sun A. Lu P. J. Zhou X. Z. Balastik M. Finn G.. (2006). The prolyl isomerase Pin1 regulates amyloid precursor protein processing and amyloid-beta production. Nature 440, 528–534. 10.1038/nature04543 16554819

[B42] Persichetti F. Trettel F. Huang C. C. Fraefel C. Timmers H. T. Gusella J. F.. (1999). Mutant huntingtin forms *in vivo* complexes with distinct context-dependent conformations of the polyglutamine segment. Neurobiol. Dis. 6, 364–375. 10.1006/nbdi.1999.0260 10527804

[B43] Pla P. Orvoen S. Saudou F. David D. J. Humbert S. (2014). Mood disorders in Huntington's disease: from behavior to cellular and molecular mechanisms. Front. Behav. Neurosci. 8:135. 10.3389/fnbeh.2014.00135 24795586 PMC4005937

[B44] Ravikumar B. Duden R. Rubinsztein D. C. (2002). Aggregate-prone proteins with polyglutamine and polyalanine expansions are degraded by autophagy. Hum. Mol. Genet. 11, 1107–1117. 10.1093/hmg/11.9.1107 11978769

[B45] Reiner A. Dragatsis I. Zeitlin S. Goldowitz D. (2003). Wild-type huntingtin plays a role in brain development and neuronal survival. Mol. Neurobiol. 28, 259–276. 10.1385/MN:28:3:259 14709789

[B46] Ross C. A. Pantelyat A. Kogan J. Brandt J. (2014). Determinants of functional disability in Huntington's disease: role of cognitive and motor dysfunction. Mov. Disord. 29, 1351–1358. 10.1002/mds.26012 25216368 PMC4197404

[B47] Ryo A. Hirai A. Nishi M. Liou Y. C. Perrem K. Lin S. C.. (2007). A suppressive role of the prolyl isomerase Pin1 in cellular apoptosis mediated by the death-associated protein Daxx. J. Biol. Chem. 282, 36671–36681. 10.1074/jbc.M704145200 17938171

[B48] Ryo A. Togo T. Nakai T. Hirai A. Nishi M. Yamaguchi A.. (2006). Prolyl-isomerase Pin1 accumulates in lewy bodies of parkinson disease and facilitates formation of alpha-synuclein inclusions. J. Biol. Chem. 281, 4117–4125. 10.1074/jbc.M507026200 16365047

[B49] Saez I. Vilchez D. (2014). The mechanistic links between proteasome activity, aging and age-related diseases. Curr. Genomics 15, 38–51. 10.2174/138920291501140306113344 24653662 PMC3958958

[B50] Sarkar S. Rubinsztein D. C. (2008). Huntington's disease: degradation of mutant huntingtin by autophagy. FEBS J. 275, 4263–4270. 10.1111/j.1742-4658.2008.06562.x 18637946

[B51] Scherzinger E. Lurz R. Turmaine M. Mangiarini L. Hollenbach B. Hasenbank R.. (1997). Huntingtin-encoded polyglutamine expansions form amyloid-like protein aggregates *in vitro* and *in vivo*. Cell 90, 549–558. 10.1016/S0092-8674(00)80514-0 9267034

[B52] Schipper-Krom S. Juenemann K. Reits E. A. (2012). The ubiquitin-proteasome system in Huntington's disease: are proteasomes impaired, initiators of disease, or coming to the rescue? Biochem. Res. Int. 2012:837015. 10.1155/2012/837015 23050151 PMC3462393

[B53] Siepe D. Jentsch S. (2009). Prolyl isomerase Pin1 acts as a switch to control the degree of substrate ubiquitylation. Nat. Cell Biol. 11, 967–972. 10.1038/ncb1908 19597489

[B54] Thompson L. M. Aiken C. T. Kaltenbach L. S. Agrawal N. Illes K. Khoshnan A.. (2009). IKK phosphorylates Huntingtin and targets it for degradation by the proteasome and lysosome. J. Cell Biol. 187, 1083–1099. 10.1083/jcb.200909067 20026656 PMC2806289

[B55] Trettel F. Rigamonti D. Hilditch-Maguire P. Wheeler V. C. Sharp A. H. Persichetti F.. (2000). Dominant phenotypes produced by the HD mutation in STHdh(Q111) striatal cells. Hum. Mol. Genet. 9, 2799–2809. 10.1093/hmg/9.19.2799 11092756

[B56] Venkatraman P. Wetzel R. Tanaka M. Nukina N. Goldberg A. L. (2004). Eukaryotic proteasomes cannot digest polyglutamine sequences and release them during degradation of polyglutamine-containing proteins. Mol. Cell 14, 95–104. 10.1016/S1097-2765(04)00151-0 15068806

[B57] Verhoef L. G. Lindsten K. Masucci M. G. Dantuma N. P. (2002). Aggregate formation inhibits proteasomal degradation of polyglutamine proteins. Hum. Mol. Genet. 11, 2689–2700. 10.1093/hmg/11.22.2689 12374759

[B58] Vilchez D. Saez I. Dillin A. (2014). The role of protein clearance mechanisms in organismal ageing and age-related diseases. Nat. Commun. 5:5659. 10.1038/ncomms6659 25482515

[B59] Wanker E. E. (2000). Protein aggregation and pathogenesis of Huntington's disease: mechanisms and correlations. Biol. Chem. 381, 937–942. 10.1515/BC.2000.114 11076024

[B60] Wheeler V. C. Auerbach W. White J. K. Srinidhi J. Auerbach A. Ryan A.. (1999). Length-dependent gametic CAG repeat instability in the Huntington's disease knock-in mouse. Hum. Mol. Genet. 8, 115–122. 10.1093/hmg/8.1.115 9887339

[B61] White J. K. Auerbach W. Duyao M. P. Vonsattel J. P. Gusella J. F. Joyner A. L.. (1997). Huntingtin is required for neurogenesis and is not impaired by the Huntington's disease CAG expansion. Nat. Genet. 17, 404–410. 10.1038/ng1297-404 9398841

[B62] Wild E. J. Tabrizi S. J. (2014). Targets for future clinical trials in Huntington's disease: what's in the pipeline? Mov. Disord. 29, 1434–1445. 10.1002/mds.26007 25155142 PMC4265300

[B63] Xu Y. X. Hirose Y. Zhou X. Z. Lu K. P. Manley J. L. (2003). Pin1 modulates the structure and function of human RNA polymerase II. Genes Dev. 17, 2765–2776. 10.1101/gad.1135503 14600023 PMC280625

[B64] Xu Y. X. Manley J. L. (2007). Pin1 modulates RNA polymerase II activity during the transcription cycle. Genes Dev. 21, 2950–2962. 10.1101/gad.1592807 18006688 PMC2049196

[B65] Yu D. Pendergraff H. Liu J. Kordasiewicz H. B. Cleveland D. W. Swayze E. E.. (2012). Single-stranded RNAs use RNAi to potently and allele-selectively inhibit mutant huntingtin expression. Cell 150, 895–908. 10.1016/j.cell.2012.08.002 22939619 PMC3444165

[B66] Zielonka D. Mielcarek M. Landwehrmeyer G. B. (2015). Update on Huntington's disease: advances in care and emerging therapeutic options. Parkinsonism Relat. Disord. 21, 169–178. 10.1016/j.parkreldis.2014.12.013 25572500

