# Supplementary material for: PIN1 Modulates Huntingtin Levels and Aggregate Accumulation: An In vitro Model
Source: Front Cell Neurosci. 2017 May 8;11:121. doi: 10.3389/fncel.2017.00121 (PMC5420564; doi:10.3389/fncel.2017.00121)
Supplement: Supplementary file 1 [file DataSheet1.docx]

Supplementary Material

**PIN1 modulates huntingtin levels and aggregate accumulation: an *in vitro* model**

**Alisia Carnemolla^1*‡^, Silvia Michelazzi^1^ and Elena Agostoni^1*^**

^1^ International School for Advanced Studies (SISSA), Area of Neuroscience, Trieste, Italy

*** Correspondence**: Alisia Carnemolla [alisia.carnemolla@kcl.ac.uk](mailto:alisia.carnemolla@kcl.ac.uk), Elena Agostoni [agostoni@sissa.it](mailto:agostoni@sissa.it)

**Supplementary Figures and Tables**

**Supplementary Figure 1. PIN1 effect is not mitigated by the length of the polyQ tract.** HEK293 cells were co-transfected with htt_1-171_Q150GFP and HA-PIN1 or pcDNA3.0. Cells were harvested for analysis 48 hours after transfection. (**A**) Representative immunofluorescent images of co-transfected cells immunostained for PIN1 (anti-HA, red) and counterstained with DAPI (blue). GFP signal (green) represents HTT. Scale bar, 20μm. Pictures were captured using a Leica CTR 6000. (**B**) mHTT aggregate amount quantified from immunostained cells as shown in (A). Data are the mean ± SEM from 4 independent experiments using 2 different batches of cells. **P<0.01. Asterisk indicates the statistically significant difference in the level of aggregates.

**Supplementary Figure 2. PIN1 overexpression reduces mHTT aggregation.** SH-SY5Y cells were co-transfected with htt_1-171_Q60GFP and HA-PIN1 or HA-PIN1DM. Cells were harvested for analysis 48 hours after transfection. (**A**) Representative immunofluorescent images of co-transfected cells immunostained for PIN1 (anti-HA, red) and counterstained with DAPI (blue). GFP signal (green) represents HTT. Scale bar, 10μm. (**B**) Representative western blot showing htt_1-171_Q60, PIN1 and β-ACTIN as loading control. Data are representative from 3 independent experiments.

**Supplementary Figure 3. PIN1 overexpression reduces huntingtin protein levels.** HEK293 cells were co-transfected with htt_1-171_Q60GFP and pcDNA3.0-HA, HA-PIN1 or HA-PIN1-DM. Cells were harvested for analysis 48 hours after transfection. Representative western blot showing htt_1-171_Q60GFP, PIN1 and β-ACTIN as loading control. Data are representative of 3 independent experiments.

**
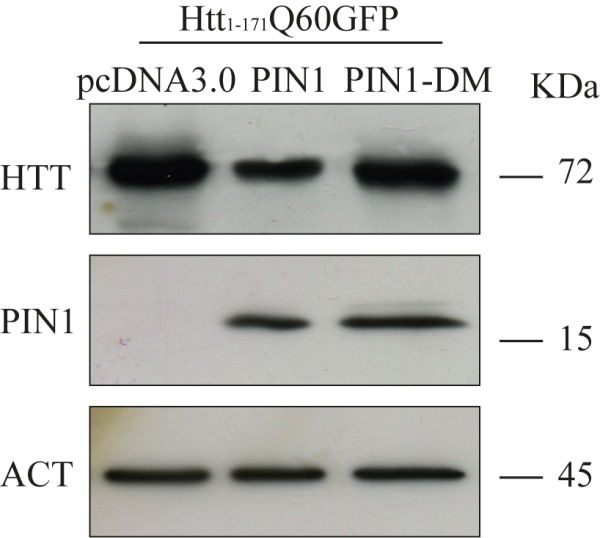
**

**Supplementary Figure 4. mHTT and YFP^u^, but not YFP, accumulate upon proteasome blockade.** HEK293 cells were transfected with the reported protein-encoding plasmids. (**A**) Cells were treated with 10μM MG-132 24 hours after transfection. Cells were harvested for analysis 6 hours after MG-132 treatment. Representative western blot showing htt_1-171_Q60GFP and β-ACTIN as loading control. (**B**) Cells were treated with 2.5μM Epoxomicin 24 hours after transfection. Cells were harvested for analysis 6 hours after Epoxomicin treatment. Representative western blot showing htt_1-171_Q60GFP, PIN1 and β-ACTIN as loading control. (**C**) Cells were transfected with the reported protein-encoding plasmids and harvested for analysis 24 hours after transfection. Representative western blot showing BECLIN1, htt_1-171_Q60GFP, PIN1 and β-ACTIN as loading control. Data are representative of 4 independent experiments. NT= non-transfected. (**D**) Representative western blot showing YFP^u^, YFP, PIN1 and β-ACTIN as loading control. Data are representative from 3 independent experiments.


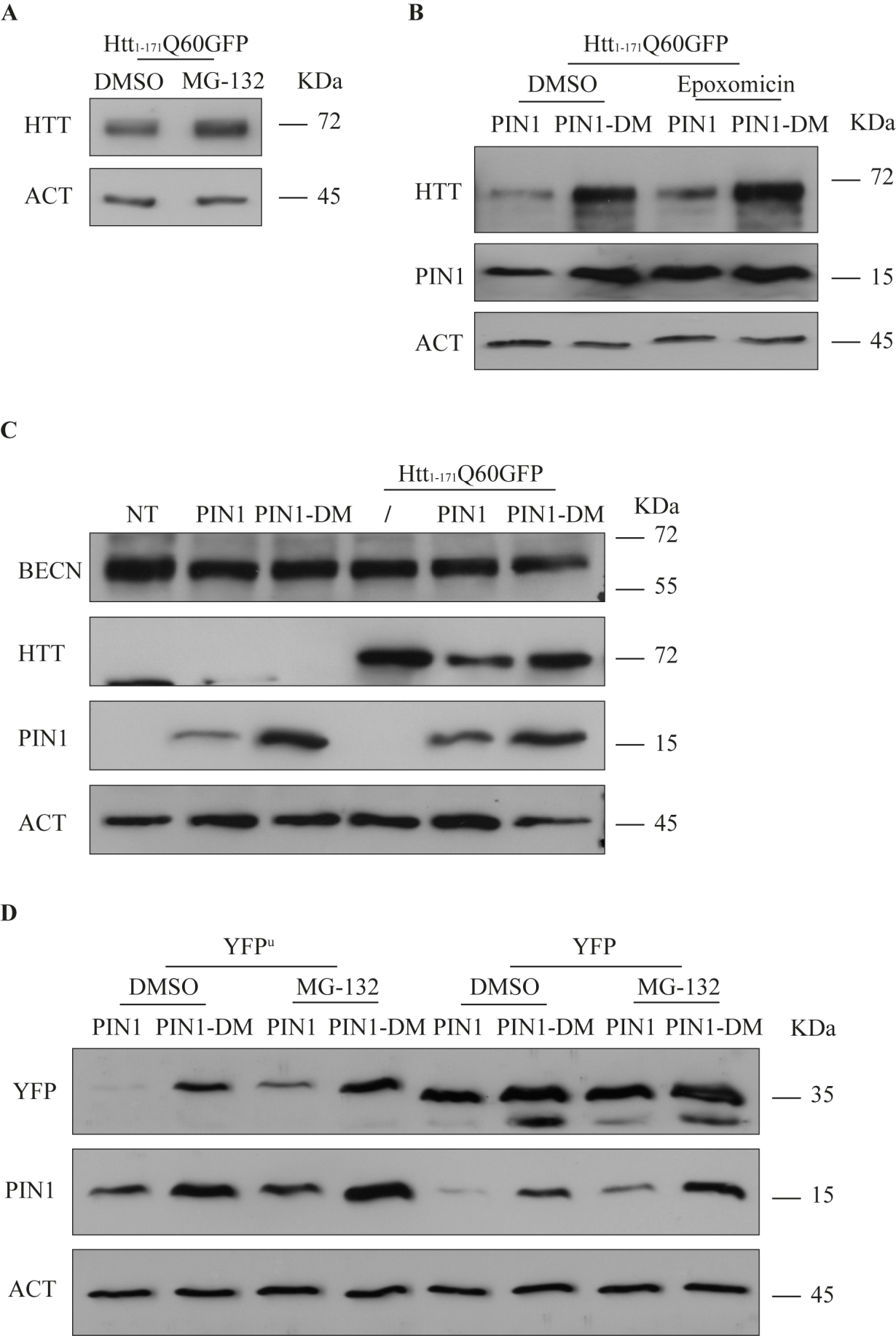


**Supplementary Table 1: RT-qPCR primers**

| **Name** | **Application** | **Farward** | **Reverse** |
| --- | --- | --- | --- |
| h*HTT* | SyBRGreen | 5’-CTACCAAGAAAGACCGTGTGAATC-3' | 5'-CCACCATCCTGACATCTGACTC-3' |
| htt_1-171_GFP | SyBRGreen | 5’-AGTGCTTTTCCAGATACCCAGAC-3' | 5'-TCCTTAAAGTCAATGCCCTTCAAC-3' |
| EGFP | SyBRGreen | 5'-GCCCGACAACCACTACCTGAG-3' | 5'- CGGCGGTCACGAACTCCAG-3' |
| hβ-actin | SyBRGreen | 5'- CGCCGCCAGCTCACCATG-3' | 5'-CACGATGGAGGGGAAGACGG-3' |
| 18S RNA | SyBRGreen | 5'- CGTCTGCCCTATCAACTTTCG-3' | 5'-GCCTGCTGCCTTCCTTGG-3' |
| *httS120A-1* | Mutagenesis | 5’-ACCGGAATTCACCATGGCGACCCTGGAAAAGCTGA-3’ | 5’-CTGAAATTCTGG**GGC**ATTT CTGACAGA-3’ |
| *httS120A-2* | Mutagenesis | 5’-TCTGTCAGAAAT**GCC**CC AGAATTTCAG-3’ | 5’-GTCATTTGCAAA ATTGCCAAAAGAAGC CA-3’ |
| *pinS67E-1* | Mutagenesis | 5’-ACGAGGATCCGCGGACGAGGAG-3’ | 5’-TGGGCCGCCGTTCCTGGCTGTG-3’ |
| *pinS67E-2* | Mutagenesis | 5’-CACAGCCAGGAACGGCGGCCCT-3’ | 5’-ATCCACTCGAGTCACTC AGTGCGGAGG-3’ |

**Supplementary Table 2: cell count, transfection efficiency and number of experiments and cell batches**

|  | **Figure 1** | | **Figure 2** | | **Figure S1** | |
| --- | --- | --- | --- | --- | --- | --- |
|  | **PIN1** | **pcDNA3.0-HA** | **PIN1** | **PIN1-DM** | **PIN1** | **pcDNA3.0-HA** |
| **# counted cells** | **1686** | **2462** | **9408** | **10793** | **903** | **1401** |
| **Transfection efficiency** | **29.8%±13%** | **29.5%±13.4%** | **31.1%±3.8%** | **27.9%±2.23%** | **52.11%±14.9%** | **48.04%±14.8%** |
| **# experiments** | **3** | **3** | **4** | **4** | **4** | **4** |
| **# cell batches** | **3** | **3** | **2** | **2** | **2** | **2** |

**Supplementary Table 3: Antibodies – source, application and working dilutions**

| **Antibody** | **Catalogue number** | **Source** | **Dilution** | **Application** |
| --- | --- | --- | --- | --- |
| HTT | MAB 5490 | Chemicon | 1:2500 | WB, FR |
| PIN1 | sc46660 (G-8) | Santa Cruz | 1:1000 | WB |
| β-ACTIN | A1978 | Sigma | 1:5000 | WB |
| GFP | GFP-1020 | Aves Lab | 1:5000 | WB |
| BECLIN1 | sc-11427 (H-300) | Santa Cruz | 1:500 | WB |
| HA | N/A | Human Hybridoma | 1:1000 | WB |
| HA | N/A | Human Hybridoma | 1:100 | ICC |

Key: WB= Western Blot; FR= Filter Retardation assay; ICC= Immunocytochemistry
